# The Relations between EGFR R521K Polymorphism and Risk of Cancer: Need for Clarification of Data in a Recent Meta-Analysis

**DOI:** 10.1155/2015/597413

**Published:** 2015-03-19

**Authors:** Jianjun Jiang, Yanjiao Li, Lifen Dai, Haiying Wu, Min Hu

**Affiliations:** ^1^The Research Center for Molecular Medicine, Kunming University, Kunming 650214, China; ^2^Department of Endocrinology, The Second Affiliated Hospital of Kunming Medical University, Kunming 650500, China; ^3^Department of Emergency and Intensive Care Unit, First Affiliated Hospital, Kunming Medical College, Kunming 650032, China

Recently we read with great interest the paper by Wang et al. [1]. The authors conducted a meta-analysis of 13 studies containing 7328 cases and 8455 controls to estimate the association of R521K polymorphism in EGFR gene with risk of cancer. This meta-analysis suggested that the EGFR R521K polymorphism is not associated with risk of cancer except a statistical difference between A and G allele frequencies in gastric cancer. It is a valuable study. Nevertheless, we would like to raise several concerns related to this article.

First, the authors made some mistakes about counting the *P* value for Hardy-Weinberg equilibrium (HWE) in control groups. According to the original data (the numbers reported by Rebai et al. [2] and Hong et al. [3] for GG/GA/AA in controls were 174/98/30 and 630/1516/770, in Table 1 in the original text), the authors calculated the *P* values through the De Finetti program (http://ihg.gsf.de/cgi-bin/hw/hwa1.pl) as 0.06 and 0.19. However, using the same program, we obtained much different results; the *P* values were equal to 0.005 and 0.02, respectively. These results significantly deviated from Hardy-Weinberg equilibrium (*P* < 0.05), and thus publication bias may be present, distorting the meta-analysis. To avoid this, the authors should reject these studies in this analysis.

Second, the racial categories of the original studies were not always clear in this paper (Table 2 in the original text). The authors pegged the Egyptians of the original study by Wang et al. as Caucasian [4]. After careful examination of the original study, we found no available data on the ethnic/racial background. Therefore, it is confusing why the authors used the country of publication as a surrogate for the ethnic background.

Finally, there are some problems of the method of stratified analyses. The authors simply categorized types into the same cancer with all races and the same race with all types of cancer (Table 2 in the original text). It is worth considering that the R521K SNP exhibits differences in allele frequencies between ethnic groups under normal conditions. Therefore, this kind of classification method only based on cancer types and ignoring differences between populations is not reliable.

In conclusion, the results of the study by Wang et al. [1] should be considered carefully. It would be valuable if the authors could provide a new, more accurate calculation after taking into account these observations.
